# Concordance in the recording of stroke across UK primary and secondary care datasets: a population-based cohort study

**DOI:** 10.3399/BJGPO.2020.0117

**Published:** 2021-02-24

**Authors:** Ann Morgan, Sarah-Jo Sinnott, Liam Smeeth, Caroline Minassian, Jennifer Quint

**Affiliations:** 1 Department of Occupational Medicine and Public Health, National Heart and Lung Institute, Imperial College London, London, UK; 2 Faculty of Epidemiology & Population Health, London School of Hygiene and Tropical Medicine, London, UK

**Keywords:** primary health care, electronic health records, Clinical Practice Research Datalink, stroke

## Abstract

**Background:**

Previous work has demonstrated that the recording of acute health outcomes, such as myocardial infarction (MI), may be suboptimal in primary healthcare databases.

**Aim:**

To assess the completeness and accuracy of the recording of stroke in UK primary care.

**Design & setting:**

A population-based longitudinal cohort study.

**Method:**

Cases of stroke were identified separately in Clinical Practice Research Datalink (CPRD) primary care records and linked Hospital Episode Statistics (HES). The recording of events in the same patient across the two datasets was compared. The reliability of strategies to identify fatal strokes in primary care and hospital records was also assessed.

**Results:**

Of the 75 674 stroke events that were identified in either CPRD or HES data during the period of the study, 54 929 (72.6%) were recorded in CPRD and 51 013 (67.4%) were recorded in HES. Two-fifths (*n* = 30 268) of all recorded strokes were found in both datasets (allowing for a time window of 120 days). Among these 'matched' strokes the subtype was recorded accurately in approximately 75% of CPRD records (compared with coding in HES); however, 43.5% of ischaemic strokes in HES were coded as 'non-specific' strokes in CPRD data. Furthermore, 48.2% had same-day recordings, and 56.2% were date-matched within ±1 day.

**Conclusion:**

The completeness and accuracy of stroke recording is improved by the use of linked hospital and primary care records. For studies that have a time-sensitive research question, the use of linked, as opposed to stand-alone, CPRD data is strongly recommended.

## How this fits in

There is an increasing focus on the use of data from routine healthcare settings to support not only clinical risk prediction, but also pragmatic clinical trials and regulatory decision making. However, in any of these research scenarios, the successful use of such data hinges on the ability to accurately identify key outcomes and prevalent comorbidities, such as stroke. This study demonstrates that reliance on a single dataset to identify stroke is likely to underestimate cases of stroke, and, for this reason, the use of linked health data is advocated, especially for research in which the timing of stroke is critical. Linkage to stroke audit data, as a means of improving knowledge of stroke epidemiology in the UK, is also recommended as a desirable long-term goal.

## Introduction

Stroke is the UK’s fourth most common cause of death^1^ and a major cause of disability.^2^ Furthermore, with costs to society totalling some £23 billion per year,^3^ stroke remains a major focus of cardiovascular research as academics, clinicians, and policymakers endeavour to better understand its epidemiology and aetiology, and so reduce its burden.

Routinely-collected data, including electronic health records (EHRs) from primary care and administrative data from hospitals, are frequently used to study stroke.^4,5^ Indeed, such data are becoming increasingly important for regulatory decision making concerning the effectiveness and cardiovascular safety of drugs, especially since traditional clinical trials are expensive, limited in their generalisability, and require long follow-up times to accrue major events such as stroke.^6,7^ Other uses of EHR data extend to clinical risk prediction^8,9^ and interventional research such as pragmatic trials.^10^ The validity of any research based on real-world data is, however, dependent on how well researchers can identify outcomes such as stroke. Several studies have revealed discrepancies between data sources in the recording of certain health outcomes, in particular acute outcomes, and have noted that reliance on just one data source risks missing a substantial proportion of cases.^11,12^

Only two studies — one on ischaemic and the other on haemorrhagic stroke — have examined the reliability of stroke recording in UK primary care databases.^13,14^ Both were conducted in the same dataset (The Health Improvement Network [THIN]) and both were limited in that, first, they recognised only hospitalised strokes, and second, they validated the diagnostic accuracy of Read-coded primary care data from within the same data source (using different data fields) rather than against another data source (for example, hospital data). Furthermore, this previous work predates the introduction of the Quality and Outcomes Framework (QOF), an incentivisation system that will have impacted on the quality of the recording of stroke in primary care data post-2004.^15^

The aim of this study was to determine how well strokes are being recorded in primary care data by comparing the recording of stroke events in the same patient across their linked primary and secondary care records. The accuracy of that recording was assessed in terms of: completeness (is the event recorded in both databases?); timing (do the event dates match?); and diagnostic accuracy (is the stroke subtype correctly specified?). How reliably mortality associated with stroke can be determined in primary care and hospital data was also examined by cross-referencing against Office for National Statistics (ONS) cause-specific mortality data.

## Method

### Data sources

The CPRD is a repository of de-identified electronic medical records from a nationally representative set of UK general practices. It holds research-quality data on demographics, health-related behaviours, test results, diagnoses, referrals, and prescriptions for >11 million people.^16^ It is one of the largest databases of longitudinal medical records from primary care globally and has been extensively used in epidemiological research.^17^

For this study, CPRD data linked to both HES and ONS mortality data were used, a linkage that is possible for approximately 50% of practices contributing to CPRD, all located in England. The HES database provides data on the primary reason for a hospital admission, as well as other diagnoses and procedures carried out during that admission. For the purposes of this study, the HES database was accorded a 'gold standard' status for identifying strokes under the assumption that the majority of strokes are identified and treated in hospitals.^18^ The ONS mortality data contain the date and cause of death for deaths registered in England.

### Study design and population

A cohort study design was used. In order to be eligible for inclusion in the study patients had to be aged ≥18 years; registered for at least 1 day at a HES or ONS-linked GP practice that contributed 'up-to-standard' data to CPRD; and have at least one record denoting a stroke in either CPRD, HES, and/or ONS during the study period, 1 January 2004 to 31 December 2016.

### Identification of stroke events

Strokes were identified in CPRD using Read codes, and in HES and ONS using International Classification of Diseases (ICD)-10 codes (see Supplementary Appendix S1). All stroke events were included, including multiple events recorded in the same individual. Further details of the strategies used to identify stroke events in each dataset, including additional exclusion criteria applied, are provided in the Supplementary material (see Supplementary Appendix S2).

### Analysis

For each data source, the number of recorded stroke events was counted, both overall and by stroke subtype. The extent to which: 1) strokes in HES occurred in CPRD; and 2) strokes in CPRD occurred in HES was assessed. Stroke recording was described as concordant (a 'match') if the CPRD stroke was ≤30 days before or ≤90 days after the HES stroke. The rationale for using a +90-day recording window was to allow for the fact that some stroke patients may remain in hospital for an extended period after their initial stroke. Consequently, their CPRD record may be significantly delayed if, for instance, the date of stroke is erroneously recorded as the date of discharge letter or only recorded when a post-hospitalisation visit to primary care occurs. Allowing a minus 30-day recording facilitated capture of stroke referrals from primary care. The degree of completeness of recording between the two data sources was reported using a Venn diagram. Sensitivity analyses explored the effect on concordance of restricting the analysis to: 1) non-fatal stroke (survival to 30 days); and 2) first-ever stroke only.

For matched strokes, the accuracy in timing was described in terms of the number of days between a recording in HES and a recording in CPRD. The level of diagnostic accuracy was assessed across the two datasets by estimating the proportion of matched strokes that were assigned the same stroke subtype.

Finally, a separate analysis was conducted in which various strategies and definitions were used to identify fatal strokes in CPRD and HES data . The ONS data were then used as a gold standard to ascertain the positive predictive value of these strategies for defining a fatal stroke.

## Results

**Figure 1.**
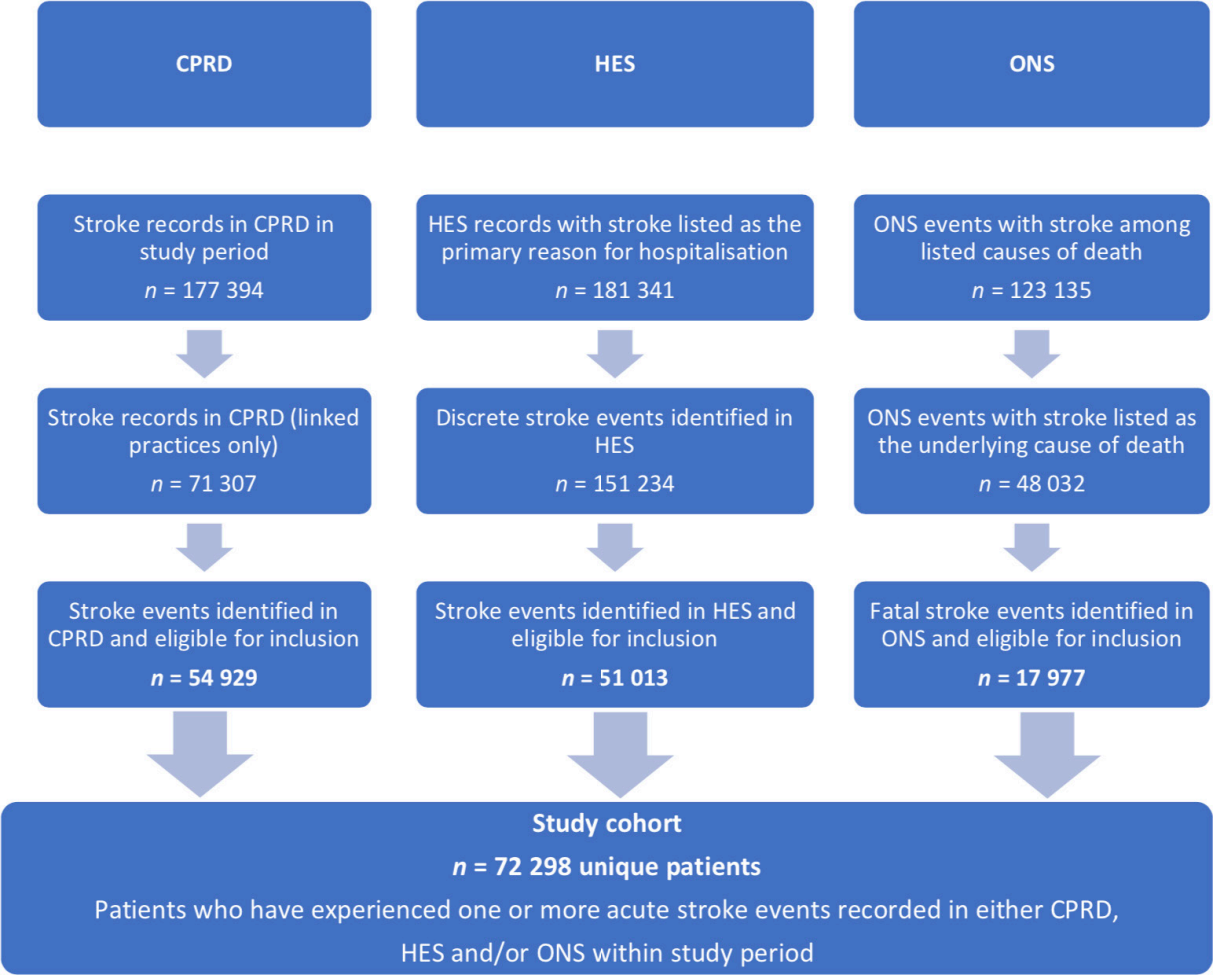
Identification of eligible stroke events in each data source. In CPRD, individual stroke records were combined into a single record, which represented a single, discrete event using the 90-day rule (see Supplementary Appendix S2). Similar criteria were used to identify separate stroke events in the same patient in HES data. Moreover, events were restricted to those that occurred in the same (that is, concurrent) periods of follow-up across the three linked data sets within the study period, 1 January 2004 to 31 December 2016. CPRD = Clinical Practice Research Datalink. HES = Hospital Episode Statistics. ONS = Office for National Statistics (mortality data).

Within the study period, a total of 72 298 adults experienced at least one stroke event that was recorded in ≥1 of the three databases: CPRD (*n* = 54 929 events), HES (*n* = 51 013 events), or ONS (*n* = 17 977 deaths) (Figure 1). In both CPRD and HES data, approximately one-fifth of all strokes were coded as haemorrhagic (16.7% in CPRD and 22.0% in HES). In contrast, 59.9% of strokes in HES were recorded as ischaemic, while only 31.0% of CPRD strokes were coded as such (Table 1). Atrial fibrillation was the most common risk factor in those who suffered a fatal stroke, while diabetes and hypertension exhibited similar prevalence across all three datasets (Table 1).

**Table 1.** Number of recorded strokes and risk factor prevalence in the three cohorts identified in CPRD, HES, and ONS data (*N* = 72 298)

**Characteristic**	**Primary care** **CPRD,^a^*n* (%)**	**Hospital admissions HES,^b^*n* (%)**	**Cause-specific mortality** **ONS, *n* (%)**
**All strokes**	54 929 (100)	51 013 (100)	17 977 (100
Ischaemic strokes	17 027 (31.0)	30 554 (59.9)	2248 (12.5)
All haemorrhagic strokes SAH Other haemorrhagic stroke^c^	9181 (16.7)2749 (5.0)6432 (11.7)	11 266 (22.0)2726 (5.3)8540 (16.7)	4289 (23.9)1123 (6.2)3166 (17.6)
Stroke, not otherwise specified	28 721 (52.3)	9193 (18.0)	11 440 (63.6)
**M** **edian a** **ge** **,** **years** **(** **IQR** **)**	76.4 (65.9–83.9)	78.5 (68.2–85.4)	83.8 (76.6–89.3)
**Sex,** **male**	27 215 (49.5)	24 308 (47.7)	6893 (38.3)
**Ethnic group**			
WhiteSouth AsianBlackOther (including Mixed)Unknown/missing	50 161 (91.3)848 (1.5)584 (1.1)494 (0.9)2842 (5.2)	46 993 (92.1)724 (1.4)571 (1.1)528 (1.0)2290 (4.5)	15 347 (85.4)148 (0.8)131 (0.7)179 (1.0)2172 (12.1)
**Family history of IHD**	8064 (14.7)	7054 (13.8)	1774 (9.9)
**Smoking status**			
Never smokedEx-smokerCurrent smokerMissing	21 871 (39.8)23 658 (43.1)8949 (16.3)451 (0.8)	20 607 (40.4)21 916 (43.0)7879 (15.4)611 (1.2)	8225 (45.8)6827 (38.0)2133 (11.9)792 (4.4)
**BMI**			
UnderweightNormalOverweightObeseMissing	1261 (2.3)15 499 (28.2)16 059 (29.2)10 629 (19.4)11 481 (20.9)	1275 (2.5)14 496 (28.4)14 074 (27.6)9252 (18.1)11 916 (23.4)	715 (4.0)4946 (27.5)3374 (18.8)1910 (10.6)7032 (39.1)
**Atrial fibrillation**	8939 (16.3)	9965 (19.5)	4069 (22.6)
**Diabetes**	9317 (17.0)	9063 (17.8)	2986 (16.6)
**H** **ypertension**	31 578 (57.5)	30 249 (59.3)	10 723 (59.6)
**Dyslipidaemia**	11 454 (20.9)	10 386 (20.4)	2896 (16.1)

Patients might be represented in ≥1 column if their stroke(s) was recorded in ≥1 data source. Age, atrial fibrillation, diabetes, hypertension, and dyslipidaemia were determined at the time of the recorded stroke. BMI and smoking status were determined at the time of the first recorded stroke (in a given dataset) and assumed to be the same for any subsequent strokes in that patient. Family history of IHD refers to a family history of myocardial infarction and angina.

BMI = body mass index. CPRD = Clinical Practice Research Datalink. HES = Hospital Episode Statistics. IHD = ischaemic heart disease. IQR = interquartile range. ONS = Office for National Statistics. SAH = subarachnoid haemorrhage.

^a^In CPRD, a total of 54 929 discrete stroke events were recorded in 49 791 individual patients. ^b^In HES, a total of 51 013 discrete stroke events were recorded in 47 481 individual patients. ^c^Includes intracerebral haemorrhagic stroke and haemorrhagic strokes not otherwise specified.

In CPRD data, just 10 individual codes accounted for 82.6% of all recorded strokes (see Supplementary Appendix S3: Table S3.1). Two non-specific codes ('cerebrovascular accident unspecified' and 'stroke and cerebrovascular attack unspecified') comprised almost 50% of all coded events. In HES data, over 90% of strokes identified were described by a set of only 10 ICD-10 codes (see Supplementary Appendix S3: Table S3.2).

### Agreement between CPRD and HES data

**Figure 2.**
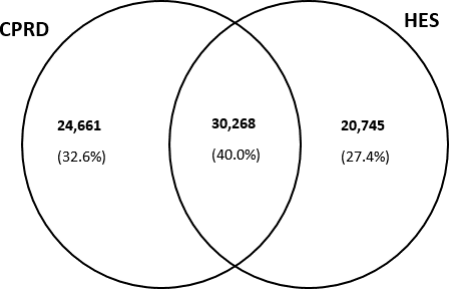
Number and percentage of all strokes (fatal and non-fatal) recorded in primary care (CPRD) and in hospital (HES) data sources (total number of recorded stroke events = 75 674). These data are based on a 120-day recording window, such that 30 268 HES-recorded stroke events had a 'matching' record in CPRD that was dated within 120 days of the date of hospital admission for stroke, either 30 days before or up to 90 days after. CPRD = Clinical Practice Research Datalink. HES = Hospital Episode Statistics.

Of 75 674 stroke events identified in either CPRD or HES data, 54 929 (72.6%) were recorded in CPRD and 51 013 (67.4%) were recorded in HES (Figure 2, Table 1). Two-fifths (*n* = 30 268) of coded strokes were 'matched strokes', that is were present in both datasets (Figure 2). Of all HES strokes, 59.3% were found in CPRD data. Of all CPRD strokes, 55.1% were found in HES data.

When the analysis was restricted to non-fatal strokes, the proportion of events reported in both datasets increased slightly to 43.3%. The proportion of hospitalisations for non-fatal strokes that were reflected in the primary care record increased to 66.4% (see Supplementary Appendix S3: Figure S3.1). However, no improvement in concordance was observed when the analysis was limited to first strokes; the proportion of 'matched' events remained at around 39.7% (see Supplementary Appendix S3: Figure S3.2).

#### Agreement in subtyping for matched strokes

Nearly three-quarters of 'matched strokes' coded as haemorrhagic in HES were also coded as haemorrhagic in CPRD (Table 2a). Likewise, 74.1% of strokes identified as haemorrhagic in CPRD were coded as such in HES data (Table 2b). In contrast, only 43.5% of ischaemic strokes in HES data were also coded as ischaemic in CPRD data. Strokes coded as ischaemic in CPRD data were confirmed as such in 85.7% of cases in HES data. A large proportion (71.0%) of strokes coded with non-specific codes in CPRD were coded as ischaemic in HES data (Table 2b).

**Table 2.** Degree of concordance in the recording of strokes by subtype across primary and secondary care data sources

a. HES-recorded strokes in CPRD
**HES-recorded strokes**	**CPRD-recorded strokes**			
	**NOS**	**Ischaemic**	**Other haemorrhagic**	**SAH**
	**(** ***n*** **=** **14** **599** **)** ***n* (%)**	**(** ***n*** **=** **9** **968** **)** ***n* (%)**	**(** ***n*** **=** **3** **999** **)** ***n* (%)**	**(** ***n*** **=** **1** **700** **)** ***n* (%)**
**NOS (** ***n*** **=** **4** **808** **)**	**3** **358** **(69.8)**	1240 (25.8)	203 (4.2)	7 (0.2)
**Ischaemic (** ***n*** **=** **19** **648** **)**	10 371 (52.8)	**8** **542** **(43.5)**	713 (3.6)	22 (0.1)
**Other haemorrhagic (** ***n*** **=** **4** **105** **)**	826 (20.1)	162 (3.9)	**2** **966** **(72.3)**	151 (3.7)
**SAH (** ***n*** **=** **1** **707** **)**	49 (2.8)	19 (1.1)	119 (7.0)	**1** **520** **(89.0)**
**b. CPRD-recorded strokes in HES**				
**CPRD-recorded strokes**	**HES-recorded strokes**			
	**NOS**	**Ischaemic**	**Other haemorrhagic**	**SAH**
	**(** ***n*** **=** **4** **808** **)** ***n* (%)**	**(** ***n*** **=** **19** **648** **)** ***n* (%)**	**(** ***n*** **=** **4** **105** **)** ***n* (%)**	**(** ***n*** **=** **1** **707** **) *n* (%)**
**NOS (** ***n*** **=** **14** **599** **)**	**3** **358** **(23.0)**	10 368 (71.0)	824 (5.6)	49 (0.3)
**Ischaemic (** ***n*** **=** **9** **968** **)**	1240 (12.4)	**8** **547** **(85.7)**	162 (1.6)	19 (0.2)
**Other haemorrhagic (** ***n*** **=** **3** **999** **)**	203 (5.1)	713 (17.8)	**2** **964** **(74.1)**	119 (3.0)
**SAH (** ***n*** **=** **1** **700** **)**	7 (0.4)	22 (1.3)	151 (8.9)	**1** **520** **(89.4)**

CPRD = Clinical Practice Research Datalink. HES = Hospital Episode Statistics. NOS = stroke not otherwise specified. SAH = subarachnoid haemorrhage.

#### Timeliness of matched strokes

Of the 30 268 CPRD–HES 'matched strokes', 48.2% (*n* = 14 587) had concordant event dates. This percentage increased to 56.2% (*n* = 17 006 strokes) when the criterion for an 'exact match' was extended to 1 day either side of a HES stroke (see Supplementary Appendix S3: Table S3.3) and increased to over 90% within 60 days of the date of hospital admission (see Supplementary Appendix S3: Figure S3.3).

### Identification of fatal strokes

A total of 17 977 individuals were identified in ONS data as having died as a result of a stroke during the study period. A quarter (23.9%) of those deaths were attributed to haemorrhagic stroke, and 12.5% to ischaemic stroke. Over half were coded using ICD-10 code I64 (stroke not otherwise specified) (Table 3).

**Table 3 .** Number of study-eligible fatal strokes recorded in ONS, CPRD, and HES data sources

	**ONS**	**CPRD**	**HES**
**Stroke subtype (ICD-10 code)**	**Patients, *n***	**Proportion of total, %**	**Patients, *n***	**Proportion of total, %**	**Patients, *n***	**Proportion of total, %**
All strokes (I60–I64)	17 977	100	5849	100	11 236	100
Ischaemic strokes (I63)	2248	12.5	822	14.1	5032	44.8
All haemorrhagic strokes (I60–l62)	4289	23.9	1434	24.5	3785	33.7
SAH (I60)	1123	6.2	387	6.6	810	7.2
Other haemorrhagic^a^ (I61–I62)	3166	17.6	1047	17.9	2975	26.5
Stroke, not otherwise specified (I64)	11 440	63.6	3593	61.4	2419	21.5

^a^Includes intracerebral haemorrhagic stroke and haemorrhagic strokes not otherwise specified. CPRD = Clinical Practice Research Datalink. HES = Hospital Episode Statistics. ICD-10 = International Classification of Diseases, version 10. ONS = Office for National Statistics. SAH = subarachnoid haemorrhage.

A total of 5849 of 54 929 CPRD-recorded strokes were categorised as fatal strokes (defined as death within 30 days after stroke), giving a stroke mortality of 10.6% in CPRD (Table 3). Extending the definition of fatal stroke to include deaths within a year increased the stroke mortality to 19.4%. In HES data, 11 236 of 51 013 strokes were categorised as being fatal, giving a stroke mortality of 22.0%.

**Figure 3.**
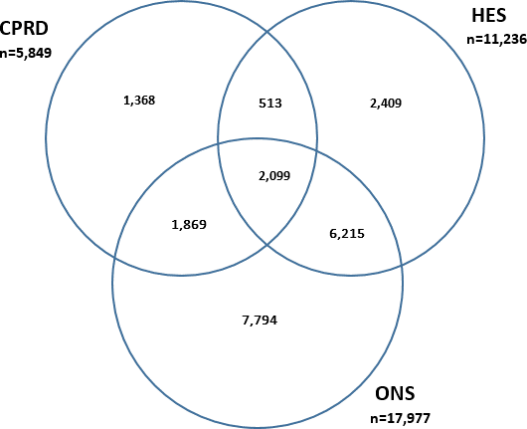
Number of fatal strokes recorded in primary care (CPRD: 30-day definition), in hospital care (HES: discharge status), and ONS (underlying cause of death). Total CPRD *n* = 54 929; total HES *n* = 51 013. CPRD = Clinical Practice Research Datalink. HES = Hospital Episode Statistics. ONS = Office for National Statistics.

The strategies for identifying fatal strokes in CPRD and HES data captured relatively few ONS-recorded events, 3968 (22.1%) and 8314 (46.2%), respectively (Figure 3). However, almost 70% of CPRD-identified fatal strokes were confirmed as such in ONS data (see Supplementary Appendix S3: Table S3.4). For HES-identified stroke deaths, the positive predictive value was better still, at around 76% (see Supplementary Appendix S3: Table S3.5).

## Discussion

### Summary

Overall, this study found a disappointingly low level of concordance in the recording of stroke events between primary care and hospital statistics. Only 40% of all identified strokes (*n* = 75 674) were captured in a timely fashion in both datasets (that is, within a timeframe of 120 days).

### Strengths and limitations

To the authors’ knowledge, this is the first UK study to cross-reference the Read-coding of stroke in UK primary care data against other data sources, at least since the introduction of QOF. It is also the first attempt to examine the reliability of strategies to identify fatal strokes in primary care and hospital data by using linked ONS data as a gold standard.

A previous study examining the concordance of MI recording in the same data sources had the advantage of being able to draw on an additional linked dataset, the national MI register (the Myocardial Ischaemia National Audit Project [MINAP]).^12,19^ Linkage to the Sentinel Stroke National Audit Programme, the national stroke registry, which contains data on around 90% of all stroke hospitalisations in England and Wales,^20^ would likewise have added to the scope of this study, in particular in terms of confirming not only the type of stroke suffered, but also the date on which it occurred.

Other study limitations stem from the inherent nature of stroke itself. Relative to other acute cardiovascular diseases, a diagnosis of stroke is more uncertain. Moreover, the experience of stroke can vary from an acute event of a few days duration to a protracted illness, with multiple sequelae and permanent disability. These factors necessitated making certain assumptions and compromises when defining appropriate time scales for distinguishing multiple events in the same patient and the CPRD–HES recording window. The choice of +90 days for both was based on clinical experience, but it is acknowledged that this may have compromised the ability to count strokes that occur in rapid succession.

### Comparison with existing literature

The findings are not dissimilar to those of a parallel study conducted for MI. The earlier MI study also identified a higher number of recorded events in CRPD (relative to HES), but the proportion of 'matched' events was higher, at around 60%.^12^

There are likely multiple reasons for the observed poor concordance in stroke recording between primary and secondary care. Strokes that occur in the community (for example, in nursing homes and never get coded in hospital data) may account for some of the discrepancy. In light of evidence that as many as 10%–16% of strokes occur in the community,^18,21^ it is certainly plausible that some strokes, in particular milder strokes and transient ischaemic attacks that are treated in the community and/or in hospital outpatient clinics, might only ever be recorded in CPRD. The possible contribution of fatal community strokes to the discordance is less certain. This is because while the primary care record will almost invariably reflect the fact that a person has died, it is less likely to document the cause of death with the result that the stroke is neither documented in CPRD nor in HES. Indeed, the general lack of coding for cause of death in CPRD, even though death occurred in hospital, may help account for the 41% of HES-recorded strokes that did not materialise in CPRD (Figure 2). Some credence to this hypothesis is provided by the results of the sensitivity analysis. Restricting the analysis to non-fatal strokes produced a small improvement in concordance, implying those who survived to 30 days post-stroke were more likely to have a corresponding CPRD record than those who did not.

While it is highly likely that a proportion of the CPRD-recorded strokes represent prevalent events (that is, are repeat codings of an earlier, as opposed to a new, event), the extent to which prevalent coding is contributing to the poor CPRD–HES overlap is also uncertain. If prevalent coding was a significant factor, the authors would have expected to see an improvement in the level of concordance when the analysis was restricted to first strokes. However, this was not the case.

Other possible reasons for the discrepancy in stroke recording between CPRD and HES relate to GP-coding practices, which are known to vary between practices.^22^ These include a failure to code the reason for a recent hospitalisation and increasing use of monitoring codes for follow-up consultations for stroke in primary care over time.^23^

This study also provided insight into the diagnostic accuracy of stroke recording in UK health databases. Of all strokes that occur in the UK, approximately 85% are ischaemic and 15% are haemorrhagic.^1^ In this analysis, 17% of CPRD strokes and 22% of HES strokes were coded as haemorrhagic, indicating some slight overrepresentation in both datasets of the latter. Conversely, ischaemic strokes were underrepresented in CPRD data, with a prevalence of just 31%. This is likely owing to widespread use of non-specific codes in primary care; 53% of matched strokes coded as ischaemic in HES data were described using non-specific codes in CPRD data. However, of the CPRD strokes that were assigned a subtype, the subtyping was mostly accurate when compared with HES strokes. For example, 80% of ischaemic strokes in CPRD were also classified as ischaemic in HES.

Estimated case-fatality rates (10.6% in CPRD data) are broadly consistent with the one-in-eight 30-day fatality estimate derived from Sentinel Stroke National Audit Programme data.^20^ The higher fatality rate in HES data in the present study (22.7%) likely reflects differences in the definition of a fatal stroke, coupled with a bias towards more severe cases of stroke in the hospital setting.

### Implications for practice

This study has raised some concerns regarding the use of simple algorithms comprised of diagnostic clinical codes for the identification of stroke in primary care records. Only 60% of all hospitalisations for stroke were reflected in the primary care record in a timely fashion (within 90 days post-stroke). Diagnostic accuracy in CPRD is also questionable, given that a high proportion of ischaemic strokes are recorded using non-specific codes. However, when a stroke is subtyped as being ischaemic or haemorrhagic in origin, the subtyping is accurate in approximately 75% of cases (relative to HES recording). Thus, while reliance on primary care data alone may be adequate for the purposes of identifying people who have had a stroke, use of HES-linked data provides greater completeness, and better information on the timing and type of stroke experienced.
